# The salary of physicians in Chinese public tertiary hospitals: a national cross-sectional and follow-up study

**DOI:** 10.1186/s12913-018-3461-7

**Published:** 2018-08-24

**Authors:** Chunyu Zhang, Yuanli Liu

**Affiliations:** School of Public Health, Chinese Academy of Medical Sciences and Peking Union Medical College, 5 Dongdansantiao, Dongcheng District, Beijing, 100730 People’s Republic of China

**Keywords:** Physician salary, Workload, Cross-sectional, Follow-up, China

## Abstract

**Background:**

Salary is perceived as a key factor affecting job satisfaction, employment, and the retention or migration of physicians within and across countries. This study aimed to describe physicians’ salary and workload and to examine the factors determining the salary of physicians in China.

**Methods:**

We conducted a self-administered, smartphone-based national survey in 136 tertiary hospitals across 31 provinces. A total of 17,615 physicians self-reported their salaries after tax and their characteristics, practice settings, and work efforts in 2015. Then, 2498 of the physicians were followed up for the second-round survey in 2016. Univariate analysis and general estimate equations were applied to evaluate the factors associated with salary.

**Result:**

In 2015, the average annual salary of the physicians was US$13,764. Physicians in eastern China earned more than those in central (*p* < 0.001) and western China (*p* = 0.002) after adjustment for locality expenditure per capita. The salary for men in 2015 was US$14,832, which was more than that for women (US$12,912; *p* < 0.001). Of the respondents, 76.3% worked more than 40 h per week. The physicians dealt with 40 patients per day on average. Consequently, 67.2% physicians spent no more than 10 min with each outpatient. After adjustments for age and management position, salary was associated with years in practice, education background, and specialty, but not with weekly work hours and gender.

**Conclusions:**

The physicians’ salaries were relatively low, and the majority of the respondents worked more than 40 h per week. Years in practice, education background, specialty, and region were associated with salary, while weekly work hours and gender were not. To better remunerate Chinese physicians, more resources are demanded, and a workload-based salary scheme should be adopted.

## Background

In China, hospitals consist of 3 types: general hospitals, traditional Chinese medicine (TCM) hospitals, and specialty hospitals, and approximately 10% of hospitals are TCM hospitals. There are approximately 383,000 TCM physicians, comprising 15% of registered physicians [1]. The majority of hospitals are public and run by the government. The routine subsidy from the government is mainly used to pay health care workers’ basic salaries in public hospitals, while the private hospitals need to pay physicians themselves, and their salaries are based on contract.

The salaries of physicians in Chinese public hospitals consist of 3 parts: basic salary, benefits, and bonuses [2]. The basic salary subsidized by the government follows a unified standard and is determined by the physician’s educational background, technical title, and years of practice. Benefits mainly refer to mandated benefits and supplemental welfare. Mandated benefits include medical insurance, endowment insurance, unemployment insurance, maternity insurance, injury insurance, and housing funding. Supplemental welfare usually consists of transportation subsidies, management allowance, and malpractice insurance, among other items. Bonuses are regarded as the majority of the salary and have 2 distribution models. One model of bonuses is bound to the profits of the department and is comprehensively adopted by hospitals. The other is the workload count model, which is a reform that was implemented in 2004. However, despite years of trial, this incentive mechanism is not popular and has been slow to be adopted [3, 4].

Salary is a central issue in health care. Salary is perceived as a key factor affecting the job satisfaction, employment, and retention or migration of physicians within and across countries [5, 6]. Lower salary is linked to physicians’ job dissatisfaction, which in turn is linked to shortages of physicians and undesirable patient outcomes [7–9]. Reports indicate that China is suffering a chronic shortage of medical doctors in certain specialties. The proportion of young physicians has decreased by approximately 10% in China in the past 10 years, while the proportion of aged physicians has increased by nearly 10% [10]. Although there is no direct evidence that the decline in numbers of physicians is caused by low job satisfaction, it has been found that job satisfaction is negatively related to the turnover intention of physicians in China; the most frequent complaints among them are the disproportionately low salary and heavy work load [11, 12]. A survey in Zhejiang province showed that the 88% of physicians who would not want their children to be doctors cited poor pay and high pressure from work as the top 2 reasons [13].

Given the challenges of recruiting and retaining of physicians for the health system and the fact that the majority of hospitals are public in China, authorities have begun to focus on physician salaries in public hospitals; however, little national salary data are available. The aims of our research were (1) to describe physicians’ salaries and workloads and (2) to explore the underlying factors that determine physician salary in Chinese public hospitals.

## Methods

### Cross-sectional samples

The cross-sectional survey was conducted in 136 tertiary hospitals across 31 provinces of China in December 2015. Multistage sampling was used in our study.

Forty-three hospitals associated with the Chinese national health and family commission were included in the sample. In addition, 1 general hospital, 1 traditional medicine hospital, and 1 specialty hospital were selected at random from each province, for a total of 136 tertiary hospitals.

We assumed that the CV (coefficient of variation) for the average annual salary was 0.8 based on the results of pilot tests, set the significance level at 0.05, and estimated a minimum sample size of 130 physicians at each hospital. To ensure that the minimum sample size was reached, we planned to select 150 physicians from each hospital. Physicians were eligible for inclusion in this study if they (1) worked full-time and (2) had practised for more than 1 year at the sampled hospital. Physicians were excluded from this study if they (1) had retired but were re-employed or (2) had previously worked as a physician but switched to full-time work as a researcher or administrator. In each sample hospital, the human resources department provided a list of the eligible physicians and categorized them into 3 groups (residents, attendings, and associate chiefs/chiefs). We selected physicians from the 3 groups proportionally according to their representation in each group. Ultimately, 20,400 physicians received invitation to take part in our survey. In total, the actual number of responding physicians was 17,975 (with a response rate of 88.11%), and the number of valid responses was 17,615. Ethics approval was obtained from the Research Ethics Committee of the Chinese Academy of Medical Science & Peking Union Medical College. A statement explaining the purpose of the study and informed consent to participate was included on the opening page of the survey. After accepting the terms of consent, the physicians were able to complete the online questionnaire.

### Follow-up samples

In December 2016, we invited the physicians surveyed in December 2015 to participate through short messages, hospital bulletins, and hospital office automation systems. A total of 2498 physicians responded.

### Survey instrument design

We implemented a self-administered questionnaire instrument using a smartphone platform (WeChat) to obtain information about the physicians’ salary after taxes, characteristics (including gender, age, education background, technical title, years in practice, and management position), practice settings (including hospital and department), and workload (weekly work hours, the number of visits per day, and consulting time). After all the questions were answered, the questionnaire could be submitted. Trained surveyors were assigned to every sample hospital to help the physicians complete the questionnaire. The same questionnaire was used in both rounds of surveys. The physicians’ salaries were converted to US$ (1 US$ = 6.47 RMB in December 2015 and 1 US$ = 6.95 RMB in December 2016).

### Statistical analysis

T-test and ANOVA analyses were used to examine whether salary was linked to region, gender, age, years in practice, education background, technical title, management position, and specialty. Generalized estimating equation analysis was used to determine which factors affected physician salary and whether the salary had changed over time after adjusting for confounders. A *p* < 0.05 was determined to be statistically significant. SAS version 9.3 (SAS Institute, Inc., NC, USA) was used to perform the statistical analyses.

## Results

### Cross-sectional study

#### Demographic characteristics of the cross-sectional study sample

The characteristics of the study sample are shown in Table 1. A total of 17,615 physicians were surveyed in our study in 2015: 7371 from eastern China, 4621 from central China, and 5623 from western China. The mean age was 37.1 years (SD: 8.2), and the average number of years in practice was 10.4 (SD: 8.7). Of the physicians, 7851 (44.6%) were male, and 9764 (55.4%) were female.

**Table 1:** Physicians’ characteristics by region in 2015

Items	Total *N* = 17,615	East *N* (%)	Central *N* (%)	West *N* (%)
Age, years				
≤ 30	4434(25.2%)	1858(25.2%)	1026(22.2%)	1550(27.6%)
31–40	7970(45.2%)	3316(45.0%)	2162(46.8%)	2492(44.3%)
41–50	3753(21.3%)	1632(22.1%)	971(21.0%)	1150(20.5%)
≥ 51	1458(8.3%)	565(7.6%)	462(10.0%)	431(7.7%)
Years in practice				
< 10	10,076(57.2%)	4188(56.8%)	2553(55.2%)	3335(59.3%)
10–19	4300(24.4%)	1848(25.1%)	1144(24.8%)	1308(23.3%)
≥ 20	3239(18.4%)	1335(18.1%)	924(20.0%)	1335(18.1%)
Education				
Bachelor’s degree and below	5145(29.2%)	1612(21.9%)	1028(22.2%)	2505(44.5%)
Master’s	8242(46.8%)	3337(45.3%)	2489(53.9%)	2416(43.0%)
Doctorate	4228(24.0%)	2422(32.9%)	1104(23.9%)	702(12.5%)
Technical title				
Resident	5589(31.7%)	2241(30.4%)	1344(29.1%)	2004(35.6%)
Attending	6122(34.8%)	2665(36.2%)	1627(35.2%)	1830(32.5%)
Associate chief	3572(20.3%)	1488(20.2%	995(21.5%)	1089(19.4%)
Chief	2332(13.2%)	977(13.3%)	655(14.2%)	700(12.4%)
Management position				
Yes	2422(13.9%)	982(13.3%)	702(15.2%)	758(13.5%)
No	15,173(86.1%)	6389(86.7%)	3919(84.8%)	4865(86.5%)
Specialty				
Internal medicine	4905(27.8%)	2098(28.5%)	1427(30.9%)	1380(24.5%)
Surgery	3303(18.8%)	1431(19.4%)	952(20.6%)	920(16.4%)
Obstetrics	2661(15.1%)	1156(15.7%)	717(15.5%)	788(14.0%)
Paediatrics	1407(8.0%)	442(6.0%)	332(7.2%)	633(11.3%)
Emergency medicine	744(8.0%)	327(4.4%)	145(3.1%)	272(4.8%)
Traditional Chinese medicine	812(4.6%)	334(4.5%)	182(3.9%)	296(5.3%)
Otolaryngology	935(5.3%)	371(5.0%)	228(4.9%)	336(6.0%)
Anaesthesiology	778(4.4%)	312(4.2%)	180(3.9%)	286(5.1%)
Ophthalmology	536(3.0%)	298(4.0%)	107(2.3%)	131(2.3%)
Stomatology	693(3.9%)	300(4.1%)	169(3.7%)	224(4.0%)
Other	841(4.8%)	302(4.1%)	182(3.9%)	357(6.3%)

#### Average annual salary

The average annual physician salary was US$13,764 in 2015. Physicians in eastern China, including some developed areas, such as Beijing, Shanghai, and Guangdong, earned more than those in central and western China (*p* < 0.001). The average annual salaries were US$18,984, US$12,840 and US$13,872 in eastern, central, and western China, respectively. After adjustment for locality expenditure per capita, the average adjusted annual salary was US$14,505 in eastern China, which is still higher than those in central China (US$12,816, *p* < 0.001) and western China (US$13,643, *p* = 0.002). The average annual salary of the male physicians was US$14,832, which was higher than that of the female physicians (US$12,912; *p* < 0.001). The top 3 highest paid specialties were ophthalmology (US$18,156), otolaryngology (US$17,664), and stomatology (US$15,852). Traditional Chinese medicine (US$ 11,748) was the specialty with the lowest annual salary, followed by paediatrics (US$12,240) and internal medicine (US$12,372; Table 2).

**Table 2:** Average annual salary by physicians’ characteristics in 2015

Item	Average annual salary (US$)	95% CI	*p* value
Region			< 0.001
East	18,984	18,768-19,200	
Central	12,840	12,636-13,056	
West	13,872	13,656-14,076	
Gender			< 0.001
Male	14,832	14,544-15,120	
Female	12,912	12,696-13,128	
Age, years			< 0.001
≤ 30	9108	8928-9288	
31–40	13,200	12,984-13,416	
41–50	18,132	17,652-18,612	
≥ 51	19,848	18,936-20,760	
Years in practice			< 0.001
< 10	11,016	10,848-11,172	
10–19	16,380	15,984-16,776	
≥ 20	18,852	15,996-19,416	
Education			< 0.001
Bachelor’s degree and below	11,676	11,412-11,940	
Master’s	12,060	11,844-12,276	
Doctorate	19,644	19,188-20,100	
Technical title			< 0.001
Resident	8940	8784-9096	
Attending	13,356	13,116-13,596	
Associate chief	17,400	16,944-17,856	
Chief	20,880	20,148-21,612	
Management position			< 0.001
Yes	20,544	19,872-21,216	
No	12,684	12,516-12,852	
Specialty			< 0.001
Internal medicine	12,372	12,084-12,648	
Surgery	14,916	14,448-15,384	
Obstetrics	13,968	13,536-14,400	
Paediatrics	12,240	11,736-12,744	
Emergency medicine	13,680	12,864-14,496	
Traditional Chinese medicine	11,748	11,004-12,492	
Otolaryngology	17,664	16,752-18,576	
Anaesthesiology	15,600	14,808-16,392	
Ophthalmology	18,156	16,644-19,668	
Stomatology	15,852	14,784-16,920	
Other	10,788	10,236-11,340	

#### Workload

The average time per week spent seeing patients was 50.5 h (SD: 10.8). Most of the physicians (76.3%) worked more than 40 h per week; of these, 62.1% worked 41–60 h per week, and 37.9% spent more than 60 h per week with patients. The average numbers of weekly hours worked were 50.6 for male physicians and 50.3 for female physician, with no significant difference between them (*p* = 0.515). The physician saw an average of 40 patients per day in clinics; 38.0% saw fewer than 20 patients per day, 39.4% saw 21 to 50 patients per day, and 22.6% saw more than 50 patients. The average consultation time was 12 min, and 67.2% physicians spent no more than 10 min with each outpatient.

### Follow-up study

#### Demographic characteristics of the follow-up study sample

To analyse the association between salary and time, 2498 physicians were followed up in December 2016. Of these, 43.3% (*n* = 1082) were male physicians, and 56.7% (*n* = 1416) were female physicians; 36.4% (*n* = 909) were from eastern China, 22.7% (*n* = 566) were from central China, and 41.0% (*n* = 1023) from western China. The average age of the physicians in the follow-up sample was 37.5 years (SD: 7.4), the average number of years in practice was 10.6 (SD: 8.2), and the average hours worked per week was 49.6 (SD: 11.1; Table 3).

**Table 3:** Characteristics of the physicians followed up in 2016

Item	Number	Percent
Gender		
Male	1080	43.2
Female	1418	56.8
Region		
East	909	36.4
Central	566	22.7
West	1023	41.0
Age, years		
≤ 30	448	17.9
31–40	1300	52.0
41–50	566	22.7
≥ 51	184	7.4
Years in practice		
< 10	1398	56.0
10–19	674	27.0
≥ 20	426	17.1
Education		
Bachelor’s degree and below	806	32.3
Master’s	1267	50.7
Doctorate	425	17.0
Technical title		
Resident	644	25.8
Attending	998	40.0
Associate chief	538	21.5
Chief	318	12.7
Management position		
Yes	420	16.8
No	2078	83.2
Specialty		
Internal medicine	568	22.7
Surgery	408	16.3
Obstetrics	425	17.0
Paediatrics	110	4.4
Emergency medicine	80	3.2
Traditional Chinese medicine	263	10.5
Otolaryngology	125	5.0
Anaesthesiology	109	4.4
Ophthalmology	63	2.5
Stomatology	54	2.2
Other	293	11.7

#### Factors influencing physician salary

The results of an analysis comparing physicians’ salaries among subgroups, adjusted for age and management position, are presented in Table 4.

**Table 4:** Results of 2498 physicians’ longitudinal analysis of factors associated with salary, 2015–2016

Items	Estimate (SE)	*p* value
Gender		
Male	Ref.	
Female	−48.6	0.1655
Age	14.0	0.0051
Years in practice	13.0	0.0014
Education		
Doctorate	Ref.	
Bachelor’s and below	− 538.0	<.0001
Master’s	−383.0	<.0001
Technical Title		
Chief	Ref.	
Resident	− 345.0	0.0008
Attending	−230.1	0.0121
Associate chief	− 142.7	0.0981
Management position		
Yes	Ref.	
No	−274.3	<.0001
Workload	−1.8	0.7399
Specialty		
Paediatrics	Ref.	
Internal medicine	− 160.1	0.0011
Surgery	11.61	0.8489
Obstetrics	33.8	0.5340
Traditional Chinese medicine	− 166.8	0.0113
Emergency medicine	−87.0	0.2086
Otolaryngology	218.5	0.0182
Anaesthesiology	191.7	0.0137
Ophthalmology	73.2	0.6268
Stomatology	38.0	0.7160
Other	−60.7	0.3862
Region		
West	Ref.	
Central	262.8	<.0001
Middle	−176.3	<.0001
Time (2015y = 1, 2016y = 2)	394.4	<.0001

Those with more years in practice (*p* = 0.001) and a higher education level (*p* < 0.001) earned more. Chief physicians had a higher salary than residents (*p* < 0.001) and attending physicians (*p* = 0.012). Compared with paediatrics, otolaryngology (*p* = 0.018) and anaesthesiology (*p* = 0.014) had relatively higher salaries, while traditional Chinese medicine (*p* = 0.011) had lower salaries. Physicians in the western part of the country earned less than those in the eastern region (*p* < 0.001) but more than those in central China (*p* < 0.001). However, there was no gender difference in salary (*p* = 0.165). The weekly number of hours worked had no effect on salary (*p* = 0.740, Table 4).

## Discussion

In this study, we conducted a national survey to analyse the average physician salary, determine physicians’ workloads, and examine the potential factors that influence physician salary in China. The average annual physicians’ salary was US$13,764, and the average number of hours worked per week was 50.5.

**Fig. 1:**
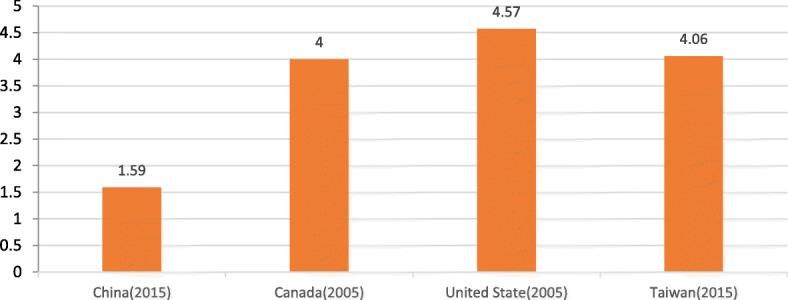
The ratio of physician salaried income to GDP per capita Source: For Canadian and US physician income, see reference [15]. For Taiwan physician salaried income, see reference [16]. For gross gemestic product per capita in those countries and regions from IMF, see reference [40]

The average annual salary (US$13,764) uncovered for Chinese physicians in our study is close to the finding reported by Dingxiangyuan ($12,360), an online forum for health care workers, although the characteristics of the participants in that survey were largely unknown [14]. However, the annual salary found in our study is far less than those reported for the United States, Canada, and Taiwan. In 2005, it has been reported that physicians earned approximately US$230,000 and US$130,000 in the United States and Canada, respectively [15]. Physicians in Taiwan, with an average annual salary of US$57,370 [16], also earned more than physicians in our survey. The gross domestic product (GDP) per capita in each country reflects the average earnings of all citizens. Figure 1 shows that the ratio of physician salaried income to GDP per capita in China is lower than those of Canada, the United States, and Taiwan. The physicians surveyed by us earned only approximately 1.5 times the Chinese GDP per capita. Canadian, US, and Taiwan physicians earned more than 4 times their local GDP per capita. The actual value in China may be lower than what we found because our data were from tertiary hospitals, which tend to have a high salary level.

More resources are demanded to better remunerate physicians, but the subsidies from the government are not sufficient. The average subsidies from the government are less than 10% of the total income of each public hospital in China [17–19]. Medicine was a main source of hospital profit in the past, but a zero-profit medicine policy has been enacted in recent years, meaning that the hospitals are faced with fewer resources to remunerate their physicians. Given that the price of health care services (such as surgery) is still far lower than medicine and material, these costs could be increased to match the value of physicians’ work and could be compensated by the social health insurance fund without increasing the patients’ out-of-pocket expenditures.

Weekly work hours were not a significant contributor to salary. However, 76.3% of the physicians in our study worked more than 40 h per week. This finding is in line with the White Paper of the Chinese Physicians’ Medical Practice Environment, in which 76.6% of physicians complained about heavy work pressure and intense workloads [20]. In China, 40 h per week is the legal work week. The average weekly worked hours was 45.5 for Chinese urban employees in 2015 [21], while the average weekly worked hours was 50.5 for physicians in our study, and 28.9% of them worked more than 60 h per week. In recent years, the number of patients treated at hospitals has increased by 59%, from 2060 million in 2009 [22] to 3270 million in 2016 [23]; this increase is attributed to universal health insurance coverage [24, 25]. The huge volume of patients has made it difficult for physicians to spend sufficient time with patients: 67.2% of the physicians in our study said they spent no more than 10 min with each outpatient, while 59% of physicians in the United States spend 13 to 24 min with each patient [26]. More time on consultation leads to better treatment decisions [27], and tense physician-patient relationships may originate from less communication [28]. Physicians are entitled to high income because of their many years of study and training, heavy responsibility, and long hours of work. Physicians have always been well paid in Canada and the United States, with their earnings reaching more than 4 times their countries’ GDP per capita [15], while those in our survey earned only 1.59 times the Chinese GDP per capita. It has been demonstrated in our study that the heavy workload and low salary of physicians are disproportionate. Given that overwork may be associated with physician burnout [29, 30] and physicians’ salaries do not compensate for the extra hours worked in China, an increased salary is a feasible way to attract and retain physicians, which would reduce workloads and provide better health care services for patients.

In our study, years of practice, educational background, technical title, management position, and specialty had effects on salary. Except for weekly work hours, as discussed above, the other factors were similar to the findings of Weeks and Wallace, who suggested that provider characteristics (including age, years in practice, and role at the hospital) and practice characteristics (including region and insurance status) were likely to affect salaries [31–34]. In terms of basic salary scheme, our study found that educational background, technical title, and years of practice were associated with physician salary; physicians with a higher level of education, a higher technical title, and more years of practice earned more. Due to the bonus payment scheme, physicians’ salaries relied heavily on departmental profits; specialty was also associated with salary because the numbers of patients seen and health care expenditures varied among specialties.

We also found that salary was unequal across regions. Physicians in eastern China earned more than those in western and central China. Although the cost of living is relatively higher in eastern China, after adjustment for locality expenditure per capita, the physician annual salary in eastern China is still higher than those in western and central China. Eastern China, which includes Beijing, Shanghai, and Guangdong, is a developed region and attracts excellent physicians for work [35, 36]. As a result, the capacity of the health care service is better in eastern China, and more severe and complex patient cases are likely to be transported there from the western and central regions to receive better treatment. As mentioned in the Introduction, the physician salary relies heavily on their departmental profit. Consequently, physicians in eastern China had the highest salaries because of the region’s high socioeconomic level and abundant patient load. These factors seem to form a circle: higher salaries attract more excellent physicians, patients flock to excellent physicians, and abundant patients lead to higher salaries. To narrow the salary difference among regions, senior specialists outreach service in poor regions for priority promotion, and ‘deals’ to pay for medical education may feasible approaches.

A gender difference has been reported in other countries, such as the United States and Japan, even after adjustment for work effort, physician characteristics, and practice characteristics [37–39]. We found that Chinese male physicians earned more than their female counterparts, but after adjustments, the salary difference became nonsignificant. In our cross-sectional sample, 31.0% and 10.7% of the female physicians worked in internal medicine and paediatrics, the lower-salary specialties, compared with 23.9% and 4.6% of their male counterparts, respectively. The proportion of male physicians (16.8%) in management positions was higher than the proportion of female physicians (11.5%). The unadjusted gender difference can be attributed to the facts that more female physicians worked in lower income specialties and that the male physicians were more likely to be in management. However, there was no difference in physician-hospital contact between genders. As a result, no adjusted gender difference was found in our study.

There are several limitations in our research. First, work hours were used to represent the work load without considering the intensity of the work. In the future, researchers hope to distinguish between academic and clinical work hours and to adjust clinical work hours using the case mix index. Second, the physicians were selected from tertiary public hospitals. We plan to survey physicians in secondary public hospitals, community health centres, village hospitals, and private hospitals in the future to form a complete picture. Third, after data from pilot tests in 3 provinces were examined, we found that the average reported salaries by specialty and technical title were near to the real levels remunerated by the hospital. Although the reported salaries were reliable, we failed to collect the grey income, such as pharmaceutical kickbacks and thanks money from patients, which are important sources of physicians’ income but are illegal in China. Compared with physicians in the west, physicians do not need to pay malpractice insurance and the annual cost of licence accreditations themselves. As a result, the income of physicians may be under estimated. Finally, there is gender difference in salary, but we do not have sufficient evidence to explain it. We plan to perform more research to determine why the difference exists.

## Conclusion

In conclusion, we found that physicians’ salaries were relatively low and that the majority of the sample worked more than 40 h per week. In addition to weekly work hours, the number of years in practice, education background, and specialty were associated with salary as a result of the salary scheme. Regional differences were found even after adjustment for locality expenditure per capita. To better remunerate Chinese physicians, more resources are demanded, and a workload-based salary scheme should be adopted.

## Abbreviations

CV: Coefficient of Variation
GDP: Gross domestic product
SD: Standard Deviation
TCM: Traditional Chinese medicine

## References

[1] National Health and Family Planning Commission PRC (2016). China health and family planning yearbook.

[2] Ran LM, Luo KJ, Wu YC, Yao L, Feng YM (2013). An analysis of China's physician salary payment system. J Huazhong Univ Sci Technolog Med Sci.

[3] Yip WC, Hsiao W, Meng Q, Chen W, Sun X (2010). Realignment of incentives for health-care providers in China. Lancet.

[4] Xia M, Pei L (2016). Research on doctor payment system of public hospitals in China. Chin Hosp.

[5] Ferrinho P, Van Lerberghe W, Julien MR, Fresta E, Gomes A, Dias F (1998). How and why public sector doctors engage in private practice in Portuguese-speaking African countries. Health Policy Plan.

[6] Morgan P, Everett CM, Humeniuk KM, Valentin VL (2016). Physician assistant specialty choice: distribution, salaries, and comparison with physicians. JAAPA JAAPA.

[7] Abelsen B, Olsen JA (2012). Does an activity based remuneration system attract young doctors to general practice?. BMC Health Serv Res.

[8] Sarma S, Devlin RA, Belhadji B, Thind A (2010). Does the way physicians are paid influence the way they practice? The case of Canadian family physicians’ work activity. Health Policy.

[9] Kahn D, Pillay S, Veller MG, Panieri E, Westcott MJ (2006). General surgery in crisis--comparatively low levels of remuneration. S Afr J Surg.

[10] Lien SS, Kosik RO, Fan AP, Huang L, Zhao X, Chang X (2016). 10-year trends in the production and attrition of Chinese medical graduates: an analysis of nationwide data. Lancet.

[11] Wang Z, Xie Z, Dai J, Zhang L, Huang Y, Chen B (2014). Physician burnout and its associated factors: a cross-sectional study in shanghai. J Occupat Health.

[12] Zhang Y, Feng X (2011). The relationship between job satisfaction, burnout, and turnover intention among physicians from urban state-owned medical institutions in Hubei. China: a cross-sectional study BMC Health Serv Res.

[13] Wu D, Wang Y, Lam KF, Hesketh T (2014). Health system reforms, violence against doctors and job satisfaction in the medical profession: a cross-sectional survey in Zhejiang Province. Eastern China BMJ Open.

[14] Ding XY (2016). Report on Chinese doctors’ salaries in 2015.

[15] Duffin J (2011). The impact of single-payer health care on physician income in Canada, 1850-2005. Am J Public Health.

[16] Bureau of Statistics of Taiwan. Taiwan statistics year-book 2016. http://www.dgbas.gov.tw/lp.asp?CtNode=3120&CtUnit=1049&BaseDSD=34 &mp=1. Accessed 29 Nov 2017.

[17] Xu Y (2016). Problems and countermeasures of public hospital fiscal input in China. Med Soc.

[18] Tan H, Yan W, Liu X, Zhu X, Zheng W (2016). Analysis on the economic operation and development status of urban public hospitals in Chongqing. Chin Health Econ.

[19] National of Health and Family Planning Commission of the People’s Republic of China (2015). Health and family planning statistic year book of China.

[20] Association CMD. The white paper of Chinese medical doctors’ practice environment. http://www.cmda.net/xiehuixiangmu/falvshiwubu/tongzhigonggao/2015-05-28/14587.html. Accessed 11 Sept 2017.

[21] Ministry of Human Resources and Social Security of the People’s Republic of China. Weekly working hours in urban area by sector and sex http://www.mohrss.gov.cn/SYrlzyhshbzb/zwgk/szrs/tongjinianjian/201803/t20180302_289122.html. Accessed 24 July 2018.

[22] National Health and Family Planning Commission of the People's Republic of China. Health statistics yearbook. 2010. http://www.nhfpc.gov.cn/htmlfiles/zwgkzt/ptjnj/year2010/index2010.html. Accessed 11 Sept 2017.

[23] National Health and Family Planning Commission of the People's Republic of China. China health and family planning development bulletin. 2017. http://www.moh.gov.cn/guihuaxxs/s10748/201708/d82fa7141696407abb4ef764f3edf095.shtml?winzoom=1. Accessed 11 Sept 2017.

[24] Meng Q, Xu L, Zhang Y, Qian J, Cai M, Xin Y (2012). Trends in access to health services and financial protection in China between 2003 and 2011: a cross-sectional study. Lancet.

[25] Chen J, Yu H, Dong H (2016). Effect of the new rural cooperative medical system on farmers’ medical service needs and utilization in Ningbo. China BMC Health Serv Res.

[26] Medscape. Medscape physician compensation report 2017. http://www.medscape.com/slideshow/compensation-2017-overview-6008547. Accessed 11 Sept 2017.

[27] de Wit NJ. A “time out consultation” in primary care for elderly patients with cancer: better treatment decisions by structural involvement of the general practitioner. Eur J Cancer Care (Engl). 2017; 10.1111/ecc.12711. 10.1111/ecc.12711 28488327

[28] Jalil A, Zakar R, Zakar MZ, Fischer F (2017). Patient satisfaction with doctor-patient interactions: a mixed methods study among diabetes mellitus patients in Pakistan. BMC Health Serv Res.

[29] Dyrbye LN, Varkey P, Boone SL, Satele DV, Sloan JA, Shanafelt TD (2013). Physician satisfaction and burnout at different career stages. Mayo Clin Proc.

[30] Hoff T, Carabetta S, Collinson GE. Satisfaction, burnout, and turnover among nurse practitioners and physician assistants: a review of the empirical literature. Med Care Res Rev. 2017;1077558717730157 10.1177/1077558717730157. 10.1177/1077558717730157 28901205

[31] Weeks WB, Wallace AE (2006). The influence of race and gender on family physician’s annual incomes. J Am Board Fam Med.

[32] Weeks WB, Wallace AE (2006). Race and gender differences in general internists’ annual incomes. J Gen Intern Med.

[33] Weeks WB, Wallace AE (2007). Differences in the annual incomes of emergency physicians related to gender. Acad Emerg Med.

[34] Weeks WB, Wallace AE (2007). Gender differences in ophthalmologists’ annual incomes. Ophthalmology.

[35] An Y (2011). Characters and improvement strategies of distribution of high - quality medical resources. Chin Heal Qual Manag.

[36] Zhang N, Sun XJ, Li C (2014). Analyzing the equity of health resources allocation in China based on Theil index. Chinese health management.

[37] Nguyen Le TA, Lo Sasso AT, Vujicic M (2017). Trends in the earnings gender gap among dentists, physicians, and lawyers. J Am Dent Assoc.

[38] Jena AB, Olenski AR, Blumenthal DM (2016). Sex differences in physician salary in US public medical schools. JAMA Intern Med.

[39] Okoshi K, Nomura K, Taka F, Fukami K, Tomizawa Y, Kinoshita K (2016). Suturing the gender gap: income, marriage, and parenthood among Japanese surgeons. Surgery.

[40] International Monetary Fund. IMF financial data by country. http://www.imf.org/en/data#imffinancial. Accessed 24 July 2017.

